# Insights into Public-Private Sector Variations in Maternal Health Care Utilization in an Urban Slum in Mumbai

**DOI:** 10.7759/cureus.104142

**Published:** 2026-02-23

**Authors:** Shwetangi R Shinde, Swati R Deshpande

**Affiliations:** 1 Community Medicine, Lokmanya Tilak Municipal Medical College and Lokmanya Tilak Municipal General Hospital, Mumbai, IND; 2 Community Medicine, King Edward Memorial Hospital Seth Gordhandas Sunderdas Medical College, Mumbai, IND

**Keywords:** cesarean section, maternal health, private sector, public health facilities, public sector

## Abstract

Introduction

Understanding variations in maternal health across public and private sectors is essential for improving service delivery. This study aimed to (i) compare maternal health service utilization across public and private facilities in an urban slum of Mumbai, and (ii) assess switching between sectors during childbirth and factors influencing it.

Methods

An analytical cross-sectional study was conducted from March 2023 to February 2024 among recently delivered women, selected through bi-monthly simple random sampling from health post and community health worker delivery records. Data were collected through household surveys and verification of health records. Variables included sociodemographic and obstetric profiles, core indicators of the antenatal care (ANC) process, delivery characteristics, and postnatal practices. Switching patterns between the registration sector and the place of delivery were assessed. Analysis included descriptive statistics, chi-square tests, and logistic regression.

Results

A total of 283 women were enrolled through systematic bi-monthly simple random sampling over the 12-month study period. Among them, 170 (60%) were registered in the public sector and 113 (40%) in the private sector. Higher maternal education (p<0.001), pre-existing medical disorders (p=0.004), and a previous cesarean section delivery (p=0.03) independently predicted private-sector registration. ANC utilization was high across both sectors, with no significant differences in core ANC components; however, Mother and Child Protection card coverage was higher in the public sector (p<0.001), while first-trimester ultrasonography was more frequent in the private sector (p=0.01). Sectoral switching between registration and delivery occurred in 31 women (11%), with no significant net shift (p=0.27). Cesarean section rates (p=0.01) and out-of-pocket expenditure (OOPE; p<0.001) were significantly higher in private facilities, whereas birth immunization (p=0.006) and exclusive breastfeeding at discharge (p<0.001) were better in public sector deliveries.

Conclusion

The study suggests that the public sector was the more frequent entry point for maternal care, while private-sector registration was associated with higher maternal education, prior cesarean section, and pre-existing medical disorders. The ANC quality was uniformly high across sectors and switching was low, driven by factors such as perceived quality of services and cost considerations among others. Private-sector deliveries had significantly higher cesarean rates and OOPE. Public facilities performed better in program linkage, newborn immunization, and exclusive breastfeeding practices. Strengthening coordination, standardizing obstetric practices, and reducing unnecessary interventions are essential to improve continuity, equity, and financial protection in urban maternal health care.

## Introduction

Maternal health is a key indicator of health system performance and equity, particularly in low-resource settings where urban populations experience overlapping social, economic, and health vulnerabilities [1]. India’s maternal health landscape is characterized by a mixed health system, where public and private sectors coexist and contribute substantially to different components of antenatal, intrapartum, and postnatal care [2,3].

Evidence from India and other low- and middle-income countries consistently demonstrates differences between public and private sector facilities in terms of health service utilization, quality of health care, and delivery practices. Public sector health facilities form the backbone of national maternal and child health programs, delivering critical services like antenatal registration, immunization, nutritional counselling, and linkage to maternity benefit schemes. They predominantly serve women from lower and middle socio-economic strata, and are valued for their affordability and subsidized care. However, challenges such as overcrowding, staff shortages, provider burnout, and variable patient-provider interactions continue to affect their utilization. Conversely, women with higher education, income, and urban residence are more likely to seek care from private sector health facilities, which are often perceived to provide better quality of health care, shorter waiting times, improved provider responsiveness, and superior physical infrastructure [4-7].

Differences in delivery characteristics between the two sectors have also been well documented. Cesarean section rates are consistently higher in private health facilities, often exceeding recommended thresholds, with several studies suggesting that non-medical factors may contribute to this trend. Public health facilities, on the other hand, demonstrate relatively lower intervention rates and stronger adherence to standardized maternal and newborn care practices [8-11].

An important but less explored dimension of maternal health care utilization is the phenomenon of switching between public and private sectors during pregnancy and delivery. Studies have shown that women may register for antenatal care (ANC) in public health facilities to access free services and monetary benefits, while opting for private health facilities for investigations and ultrasonography. Factors influencing such switching include perceived quality of health care, cost considerations, staff behavior, waiting times, referral practices, and familiarity with the facility. This pluralistic utilization pattern can result in fragmented care, increased health expenditure, and inconsistent maternal and newborn health practices, affecting the continuity of care across sectors [2,4,12].

Although previous studies have examined differences in maternal health service utilization, community-based evidence from urban slum settings that simultaneously assesses maternal health services, delivery characteristics, postnatal practices, and sector-switching behavior remains limited. Context-specific data from such settings are required to better understand care-seeking patterns and inform efforts to improve coordination across sectors. Hence, this study was undertaken to (i) compare maternal health service utilization across public and private facilities in an urban slum of Mumbai, and (ii) assess switching between sectors during childbirth and the factors influencing it.

This article was previously presented as an oral paper on the 6th of February, 2026 at the 27th Maharashtra Joint Annual Conference of Indian Association of Preventive and Social Medicine and Indian Public Health Association - MHIAPSMIPHACON 2026.

## Materials and methods

Study setting

The study was conducted among women residing in the urban field practice area of a tertiary healthcare teaching institution in Mumbai, Maharashtra. The study area is characterized by high population density and dependence on both public and private health facilities for maternal health services.

Timeline

The study was conducted from March 2023 to February 2024.

Study design

An analytical cross-sectional study design was adopted.

Study population

The reference population included all recently delivered women residing in urban slums across Mumbai and similar metropolitan areas in India, such as Delhi, Kolkata, Chennai, etc. The study population comprised women who had delivered a live infant within the past two months and had resided in the center's catchment area for at least six months before delivery. Only live births were included to ensure comparability in delivery characteristics and postnatal practices, and inclusion was limited to deliveries within two months, which reduced recall bias and ensured access to medical records.

Sampling

The sample size was calculated using the formula for cross-sectional studies with a finite population.

$n = \frac{Nz^{2}pq}{e^{2}(N-1)+z^{2}pq}$

The prevalence (p) was taken as 56%, representing the proportion of deliveries occurring in the public sector as reported for the Mumbai district in NFHS-5 [13]. An absolute precision of 5% was used. The initial sample size of 379 was adjusted using a finite population correction, as the estimated annual number of pregnancies in the study area was 1105, calculated from the mid-year population (n=1,22,778), a crude birth rate of nine per 1,000 population, and a 10% pregnancy wastage factor. These values were obtained from the local health post data, that is updated by the Public Health Department every year. The final sample size was 283 women.

Sampling technique

A simple random sampling technique was employed using a rolling sampling frame. Every two months, a list of women who had delivered within the preceding two months was prepared using records from the health post and community health workers, constituting the sampling frame for those two months. Eligible participants were selected using simple random sampling. On average, 20-25 women were selected per two-month cycle, depending on the total number of deliveries recorded during that period. This process was repeated over the course of one year until the required sample size was achieved.

Data collection instrument and variables

Data were collected using a validated, pre-tested structured questionnaire developed in English and translated into the local languages (Hindi and Marathi). The questionnaire was designed to capture the following domains: sociodemographic variables such as age, religion, years of schooling, maternal employment status, type of family, and per capita income of the family, obstetric risk-related variables, such as parity, pre-existing medical disorders, complications in the previous pregnancy, and previous cesarean section, ANC-service related variables such as place of registration, time of registration, number of antenatal visits, and coverage of ANC core processes, delivery characteristics such as place and mode of delivery, low birth weight, birth immunization, early initiation of breastfeeding, exclusive breastfeeding at discharge, and newborn complications, health expenditure (out-of-pocket expenditure or OOPE) for delivery care and sector switching (discordance between the place of antenatal registration and the place of delivery), and reasons for switching where applicable. Medical records, discharge summaries, and maternal health cards were reviewed wherever available to verify self-reported information.

Operational definitions

Public health facilities were the ones owned and operated by the Government/Municipal Corporation, while private health facilities were the ones owned and operated by private individuals or organizations [11]. Breastfeeding initiated within one hour of birth was categorized as early initiation of breastfeeding [14].

Data collection techniques

Eligible women were visited at their homes. Interviews and record verification took 20-30 minutes per participant. Data completeness was checked before leaving each household.

Data management and statistical analysis

Data were entered into Microsoft Excel 2024 version 16.88 (Microsoft Corp., Redmond, WA, USA). Unique identifiers were used to maintain confidentiality. Statistical analysis was done using R version 4.5.2 (R Foundation for Statistical Computing, Vienna, Austria). Categorical variables were compared between public and private sector users using the Chi-Square's test or Fisher’s exact test, as applicable. Continuous variables were analyzed using the Wilcoxon rank-sum test for non-parametric data. Discordance between the sector of registration and the sector of delivery was assessed using McNemar’s test. Bivariate analysis was conducted to identify factors associated with private sector registration, and variables with a p-value <0.05 were subsequently included in the multivariable logistic regression model.

Ethical considerations

The study was conducted in accordance with the ethical principles outlined in the Declaration of Helsinki and adhered to the Indian Council of Medical Research (ICMR) National Ethical Guidelines for Biomedical and Health Research Involving Human Participants (2017). Ethical approval was obtained from the Institutional Ethics Committee - II, Relating to Biomedical and Health Research (BHR), Seth GS Medical College and KEM Hospital, Mumbai (approval no.: IEC(II)/OUT/66/2023). Written informed consent was obtained. Privacy and confidentiality were maintained, and all data were stored in password-protected files accessible only to the investigators.

## Results

**Figure 1:**
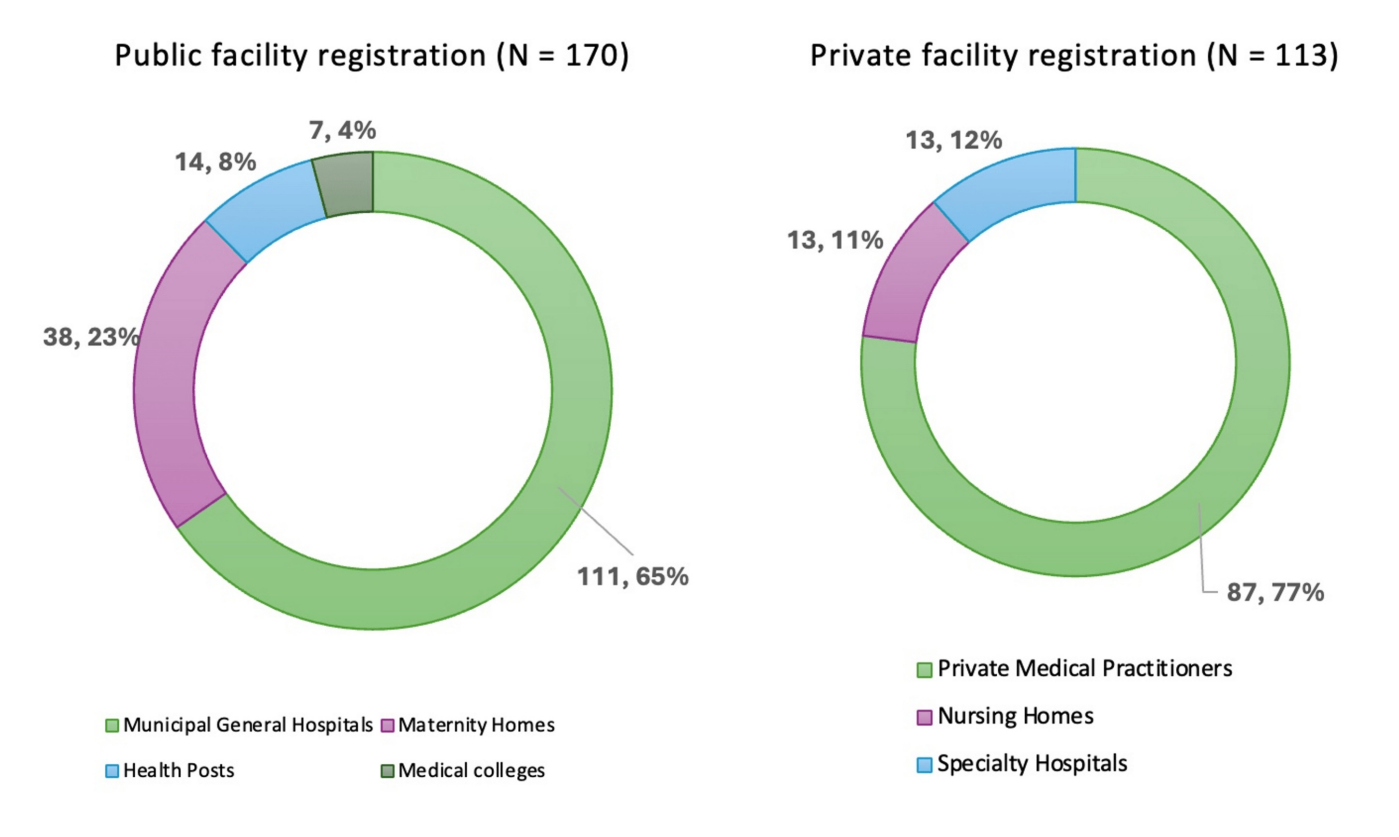
Type of health facility chosen by participants for pregnancy registration across public and private sectors (n=283)

A total of 283 recently delivered women were included in the study. Of these, 170 (60%) were registered in public-sector facilities and 113 (40%) in private-sector facilities. Within the public sector, most registrations occurred at municipal general hospitals (n=111, 65%), followed by health posts (n=38, 23%) and maternity homes (n=14, 8%). In the private sector, the majority of women were registered with private medical practitioners (n=87, 77%), followed by nursing homes (n=13, 12%) and specialty hospitals (n=13, 11%) (Figure 1).

Table 1 shows the sociodemographic and obstetric factors associated with the choice of sector for pregnancy registration.

**Table 1:** Sociodemographic and obstetric factors associated with choice of sector for pregnancy registration (n=283) *n=149 multiparous pregnancies.

Variable	Public (N=170); n (%)	Private (N=113); n (%)	Total (N=283); n (%)	Chi-square statistic and df	p-value
Age group					
<25 years	72 (42.4%)	39 (34.5%)	111 (39.2%)	χ² = 3.68, df = 2	0.16
25-29 years	58 (34.1%)	36 (31.9%)	94 (33.2%)		
≥30 years	40 (23.5%)	38 (33.6%)	78 (27.6%)		
Total	170 (60.1%)	113 (39.9%)	283 (100.0%)		
Religion					
Muslim	140 (82.4%)	89 (78.8%)	229 (80.9%)	χ² = 0.36, df = 1	0.55
Non-Muslim	30 (17.6%)	24 (21.2%)	54 (19.1%)		
Total	170 (60.1%)	113 (39.9%)	283 (100.0%)		
Years of schooling					
<5 years	40 (23.5%)	9 (8.0%)	49 (17.3%)	χ² = 27.28, df = 3	<0.001
5-9 years	76 (44.7%)	35 (31.0%)	111 (39.2%)		
10-11 years	27 (15.9%)	28 (24.8%)	55 (19.4%)		
>12 years	27 (15.9%)	41 (36.3%)	68 (24.0%)		
Total	170 (60.1%)	113 (39.9%)	283 (100.0%)		
Employment status of mother					
Employed	5 (3.0%)	9 (8.0%)	14 (5.0%)	χ² = 2.65, df = 1	0.10
Unemployed	165 (97.0%)	104 (92.0%)	269 (95.0%)		
Total	170 (60.1%)	113 (39.9%)	283 (100.0%)		
Type of family					
Nuclear	82 (48.2%)	49 (43.4%)	131 (46.3%)	χ² = 0.47, df = 1	0.49
Non-nuclear	88 (51.8%)	64 (56.6%)	152 (53.7%)		
Total	170 (60.1%)	113 (39.9%)	283 (100.0%)		
Per-capita income					
<4000 INR	87 (51.2%)	43 (38.0%)	130 (45.9%)	χ² = 4.19, df = 1	0.04
>4000 INR	83 (48.8%)	70 (62.0%)	153 (54.1%)		
Total	170 (60.1%)	113 (39.9%)	283 (100.0%)		
Parity					
Primipara	80 (47.1%)	54 (47.8%)	134 (47.4%)	χ² = 0, df = 1	1
Multipara	90 (52.9%)	59 (52.2%)	149 (52.6%)		
Total	170 (60.1%)	113 (39.9%)	283 (100.0%)		
Pre-existing medical disorders					
Yes	32 (18.8%)	33 (29.2%)	65 (23.0%)	χ² = 3.57, df = 1	0.06
No	138 (81.2%)	80 (70.8%)	218 (77.0%)		
Total	170 (60.1%)	113 (39.9%)	283 (100.0%)		
Complications in the previous pregnancy*					
Yes	36 (40.0%)	33 (59.3%)	71 (47.6%)	χ² = 4.59, df = 1	0.03
No	54 (60.0%)	80 (40.7%)	78 (52.4%)		
Total	90 (60.4%)	59 (39.6%)	149^2^ (100.0%)		
Previous cesarean section*					
Yes	11 (12.2%)	19 (32.2%)	30 (20.1%)	χ² = 7.65, df = 1	0.006
No	79 (87.8%)	40 (67.8%)	119 (79.9%)		
Total	90 (60.4%)	59 (39.6%)	149^2^ (100.0%)		

Age distribution, religion, parity, family type, and maternal employment status were comparable across sectors. However, years of schooling and per-capita income differed significantly, with a higher proportion of women registered in the private sector having completed more than 12 years of schooling (p<0.001) and reporting higher per-capita income (p=0.04). Women with pre-existing medical disorders were more likely to be registered in the private sector, although the association did not reach statistical significance (p=0.06). Among multiparous women, a history of previous pregnancy complications (p=0.03) and previous cesarean section (p=0.006) was also significantly associated with private sector registration.

Table 2 presents the bivariate and multivariable logistic regression analysis of factors associated with private sector pregnancy registration.

**Table 2:** Bivariate and multivariate logistic regression analysis of factors associated with private sector pregnancy registration (n=283) †Previous pregnancy complications and previous cesarean sections were analyzed among multiparous women only.

Variable	Category	Crude OR (95% CI)	p-value	Adjusted OR (95% CI)	p-value
Age group	<25 years	1.0 (Ref)	—	—	—
	25–29 years	1.15 (0.65–2.02)	0.64	—	—
	≥30 years	1.75 (0.97–3.14)	0.06	—	—
Religion	Muslim	1.0 (Ref)	—	—	—
	Non-Muslim	1.26 (0.69–2.29)	0.45	—	—
Years of schooling	<5 years	1.0 (Ref)	—	1.0 (Ref)	—
	5–9 years	2.05 (0.90–4.67)	0.09	2.24 (0.99–5.53)	0.06
	10–11 years	4.61 (1.88–11.31)	<0.001	5.17 (2.08–13.86)	<0.001
	>12 years	6.75 (2.82–16.16)	<0.001	7.18 (2.95–18.98)	<0.001
Employment status	Unemployed	1.0 (Ref)	—	—	—
	Employed	2.86 (0.93–8.79)	0.07	—	—
Per-capita income	<4000 INR	1.0 (Ref)	—	1.0 (Ref)	—
	≥4000 INR	1.71 (1.05–2.80)	0.03	1.41 (0.83–2.42)	0.21
Parity	Primipara	1.0 (Ref)	—	—	—
	Multipara	0.97 (0.60–1.58)	0.90	—	—
Pre-existing medical disorders	No	1.0 (Ref)	—	1.0 (Ref)	—
	Yes	1.78 (1.02–3.10)	0.04	2.53 (1.35–4.83)	0.004
Previous pregnancy complications†	No	1.0 (Ref)	—	—	—
	Yes	1.44 (0.81-2.57)	0.21	—	—
Previous cesarean section†	No	1.0 (Ref)	—	1.0 (Ref)	—
	Yes	2.56 (1.13-5.80)	0.02	2.68 (1.12-6.67)	0.03

In the bivariate analysis, higher maternal education (p<0.001), higher per-capita income (p=0.03), pre-existing medical disorders (p=0.04), and previous cesarean section (p=0.02) were significantly associated with private sector registration. Multivariate logistic regression analysis showed that maternal education demonstrated a strong independent association with private sector registration. Women with >12 years of schooling had significantly higher odds of registering in the private sector compared to those with lower education (adjusted odds ratio or AOR: 7.18; 95% CI: 2.95-18.98; p<0.001). The presence of pre-existing medical disorders (AOR: 2.53; 95% CI: 1.35-4.83, p=0.004) and previous cesarean section (AOR: 2.68; 95% CI: 1.12-6.67) were also independently associated with private sector registration. Per-capita income and previous pregnancy complications were not independently associated with private sector registration after controlling for other factors.

Table 3 shows the ANC utilization by sector of registration.

**Table 3:** Antenatal care (ANC) utilization and quality by sector of registration (n=283) *Chi-square Test; **Fisher’s Exact Test; MCP: Mother and Child Protection; HIV: Human Immunodeficiency Virus; IFA: Iron and folic acid; Td: Tetanus-Diphtheria.

ANC service indicator	Public (N=170); n (%)	Private (N=113); n (%)	Total (N=283); n (%)	Test	p-value
First trimester registration	115 (67.7%)	88 (77.9%)	203 (71.7%)	χ² = 3.02, df = 1	0.08^*^
MCP card received	138 (81.2%)	49 (43.4%)	187 (66.1%)	χ² = 41.63, df = 1	<0.001^*^
≥4 antenatal visits	106 (93.8%)	160 (94.1%)	266 (80.9%)	χ² ≈ 0, df = 1	1^*^
Hemoglobin checked	169 (99.4%)	112 (99.1%)	281 (99.3%)	Fisher’s exact test	1^**^
Weight checked	168 (98.8%)	108 (95.6%)	276 (97.5%)	Fisher’s exact test	0.12^**^
Blood pressure checked	169 (99.4%)	111 (98.2%)	280 (98.9%)	Fisher’s exact test	0.57^**^
Abdominal examination done	169 (99.4%)	111 (98.2%)	280 (98.9%)	Fisher’s exact test	0.57^**^
HIV testing done	170 (100%)	112 (99.1%)	282 (99.7%)	Fisher’s exact test	0.40^**^
Urine testing done	168 (98.8%)	108 (95.6%)	276 (97.5%)	Fisher’s exact test	0.12^**^
IFA supplementation initiated	164 (96.5%)	108 (95.6%)	272 (96.1%)	Fisher’s exact test	0.76^**^
Adequately immunized for Td	167 (98.2)	109 (96.5)	276 (97.5)	Fisher’s exact Test	0.25^**^
First trimester ultrasound done	109 (64.1%)	89 (78.8%)	198 (70.0%)	χ² = 6.25, df = 1	0.01^*^

Overall utilization was high, with 203 women (72%) registering in the first trimester and 266 (81%) completing four or more antenatal visits, without significant sectoral differences. Coverage of essential ANC components, including hemoglobin estimation, blood pressure measurement, weight monitoring, abdominal examination, Human Immunodeficiency Virus (HIV) testing, urine testing, iron and folic acid (IFA) supplementation, and tetanus-diphtheria (Td) immunization, exceeded 95% in both sectors, with no statistically significant differences. However, receipt of the Mother and Child Protection (MCP) card was significantly higher among public sector registrations (n=138, 81%) compared to the private sector (n=49, 43%) with p<0.001, while first-trimester ultrasonography was more frequently reported among private sector registrations (n=89, 79%) as compared to public sector registrations (n=109, 64%) with a p-value of 0.01. 

Table 4 presents the transition between the sector of pregnancy registration and the sector of delivery.

**Table 4:** Transition between the sector of pregnancy registration and the sector of delivery (n=281) *N=281, because there were 281 institutional deliveries out of 283; **McNemar’s Chi-square test.

Sector of delivery	Public (N=175); n (%)	Private (N=106); n (%)	Total (N=281*); n (%)	Test	p-value
Sector of registration					
Public	157 (89.7%)	11 (10.4%)	168 (59.8%)	McNemar χ² = 1.24, df = 1	0.27^**^
Private	18 (10.3%)	95 (89.6%)	113 (40.2%)		
Total	175 (62.3%)	106 (37.7%)	281 (100.0%)		

**Figure 2:**
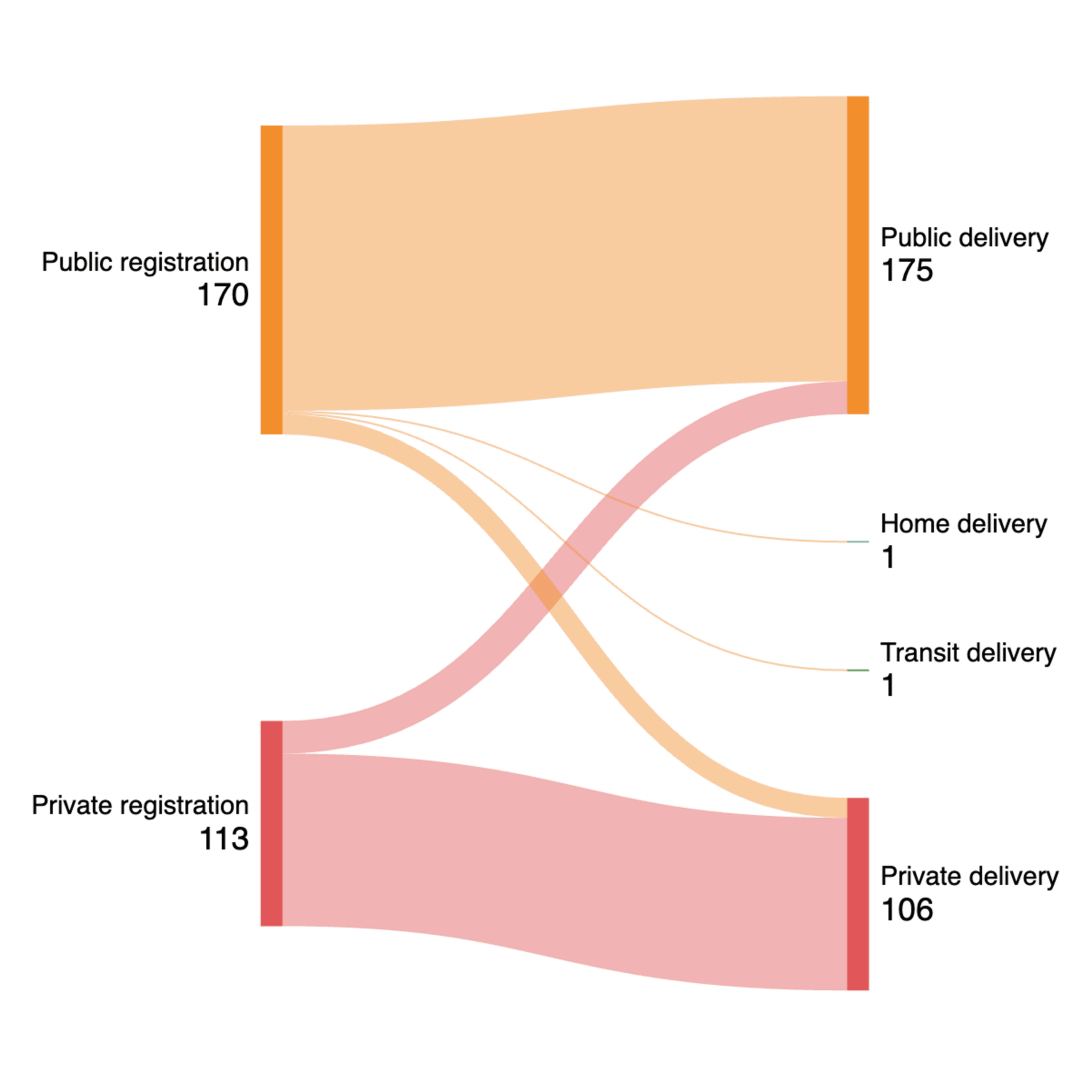
Sankey diagram showing the sectoral switching from antenatal registration to delivery (n=283) Image credit: Diagram created by using with SankeyMATIC (https://sankeymatic.com/).

Analysis was restricted to 281 women after excluding two cases of non-institutional delivery that could not be assigned to either sector. Of these, 175 (62%) deliveries occurred in public sector facilities and 106 (38%) in private sector facilities. Sectoral switching between registration and delivery occurred in 31 (11%) of cases (Table 4; Figure 2).

The reasons for switching were multifactorial. Among women who shifted from public sector registration to private sector delivery (11 of 283, 4%), the most commonly reported reasons included perceived better quality of care, greater trust in the provider, cleaner facilities, availability of doctors at the time of delivery, shorter waiting times, preference for evening outpatient services and better interpersonal behavior of staff in private facilities. Conversely, women who shifted from private sector registration to public sector delivery (18 of 283, 6.4%) cited referral for complications, particularly in the context of high-risk pregnancies, as a major reason for switching. Cost considerations also influenced this transition. There were two non-institutional deliveries, with one woman delivering at home due to the fear of a cesarean section and the other delivering in an autorickshaw on the way to the hospital. However, the McNemar’s test did not demonstrate a statistically significant overall directional shift between sectors (p=0.27), indicating bidirectional movement rather than migration towards one sector.

Table 5 shows the delivery characteristics and postnatal practices stratified by sector of delivery.

**Table 5:** Delivery characteristics and postnatal practices by sector of delivery (n=281) *N=281, because there were 281 institutional deliveries out of 283; **Chi-square test; ***Fisher’s Exact test; BCG: Bacillus Calmette-Guérin; OPV: Oral Poliovirus Vaccine.

Variable	Public (N=175); n (%)	Private (N=106); n (%)	Total (N=281)*; n (%)	Test	p-value
Mode of delivery					
Normal	110 (62.9%)	50 (47.2%)	160 (56.9%)	χ² = 6.00, df = 1	0.01^**^
Cesarean section	65 (37.1%)	56 (52.8%)	121 (43.1%)		
Total	175 (62.3%)	106 (37.7%)	281 (100.0%)		
Birth weight of the baby					
Low birth weight	59 (34.9%)	32 (29.3%)	91 (32.7%)	χ² = 0.21, df = 1	0.65^**^
Normal	116 (64.0%)	74 (69.8%)	190 (66.2%)		
Total	175 (62.3%)	106 (37.7%)	281 (100.0%)		
Birth immunisation given					
Only BCG vaccine	2 (1.5%)	7 (9.6%)	9 (4.3%)	Fisher’s Exact test	0.006^***^
BCG + OPV zero dose	31 (22.6%)	21 (28.8%)	52 (24.8%)		
All three birth doses	104 (75.9%)	45 (61.6%)	149 (71.0%)		
Not documented	38	33	71		
Total	175 (62.3%)	106 (37.7%)	281 (100.0%)		
Early initiation of breastfeeding					
Yes	112 (64.0%)	64 (60.4%)	176 (62.6%)	χ² = 0.23, df = 1	0.63^**^
No	63 (36.0%)	42 (39.6%)	105 (37.4%)		
Total	175 (62.3%)	106 (37.7%)	281 (100.0%)		
Exclusive breastfeeding at discharge					
Yes	147 (84.0%)	54 (50.9%)	201 (71.5%)	χ² = 33.82, df = 1	<0.001^**^
No	28 (16.0%)	52 (49.1%)	80 (28.5%)		
Total	175 (62.3%)	106 (37.7%)	281 (100.0%)		
Neonatal complications					
Yes	49 (28.0%)	40 (37.7%)	89 (31.7%)	χ² = 2.46, df = 1	0.12^**^
No	126 (72.0%)	66 (62.3%)	192 (68.3%)		
Total	175 (62.3%)	106 (37.7%)	281 (100.0%)		

Mode of delivery differed significantly by sector of delivery (p=0.01), with cesarean section rates being higher in the private sector (n=56, 53%) compared to the public sector (n=65, 37%). There were no significant sectoral differences in the birth weight of the newborns, with 91 (33%) low-birth-weight infants overall. Early initiation of breastfeeding was also comparable across sectors, with a total proportion of 176 (63%). However, birth vaccination coverage and exclusive breastfeeding at discharge were significantly higher among public sector deliveries (p=0.006 and p<0.001, respectively). Neonatal complications were more frequently reported among private sector deliveries, although this difference did not reach statistical significance (p=0.123).

**Figure 3:**
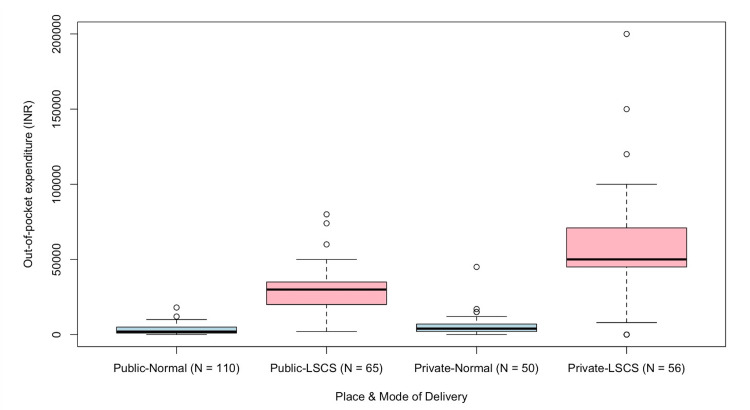
Out-of-pocket expenditure (OOPE) during delivery (in INR) by place and mode of delivery (n=281) LSCS: Lower Segment Cesarean Section.

OOPE varied markedly by both sector and mode of delivery (Figure 3), with private sector cesarean sections incurring the highest costs.

Kruskal-Wallis test confirmed a statistically significant difference in median OOPE across groups (p<0.001), indicating a substantial variation in health expenditure by both sector and mode of delivery.

## Discussion

This study offers an insight into the public-private variation in maternal health care utilization, delivery characteristics, and postnatal practices in an urban slum setting. Public sector registrations were more frequent (n=170, 60%), suggesting that public health facilities serve as the primary entry point for ANC in our setting due to affordability, familiarity, and the active role of field workers in facilitating antenatal registration. Similar patterns have been reported in Mumbai slums by More et al. [15], but studies from other urban settings have demonstrated a greater preference for the private sector, such as 70% reported by Kumar et al. [16] in Mangalore. Summan et al. [6] and Demeke et al. [5] also reported that urban residence significantly increased the likelihood of private facility use for ANC. However, these analyses did not differentiate between slum and non-slum populations. The predominance of public sector registration observed in the present study, particularly at municipal general hospitals, reflects the central role of urban public health infrastructure in addressing the maternal health needs of economically vulnerable slum communities.

The choice of sector for pregnancy registration in this study was driven primarily by socioeconomic position and perceived clinical risk rather than demographic characteristics. Higher maternal education emerged as the strongest independent predictor of private sector registration, consistent with findings from earlier studies [17]. Summan et al. [6] identified maternal education as a key determinant of private facility use, while Demeke et al. [5] also reported a higher likelihood of private sector registration among literate women. The association of pre-existing medical disorders and previous cesarean section delivery with private sector registration suggests selective utilization of private facilities by women perceiving a need for closer monitoring or specialist care. Although previous studies have reported associations between private sector utilization and higher income, lower parity, and non-Muslim religion, these variables were not significant in the adjusted analysis in the present study [5,6,12,18].

ANC utilization was high across both sectors, with 203 (70%) women registering in the first trimester and 266 (81%) completing four or more ANC visits, figures that compare favorably with national National Family Health Survey, Round 5 (NFHS-5) estimates (76% and 68%, respectively) and exceed those reported for the Mumbai district (58% and 72%, respectively) [13,19]. Despite similar overall utilization, sectoral differences were observed in specific components of care: MCP card coverage was substantially higher in the public sector (n=138, 81% vs. n=49, 43%), reflecting stronger integration with national maternal health programs and the promptness of frontline health workers. Conversely, first-trimester ultrasonography was more frequent among private sector registrations (n=89, 79% vs. n=109, 64%), consistent with prior studies attributing this to greater diagnostic availability, less waiting time, and better responsiveness [2,6,20,21]. Notably, core process indicators of ANC quality, including hemoglobin estimation, blood pressure and weight measurement, abdominal examination and HIV and urine testing, showed uniformly high coverage in both sectors (>95%). This suggests a degree of standardization in essential ANC practices, and challenges the assumption that public sector ANC is intrinsically inferior in quality for essential ANC processes [12,22,23].

Most women delivered in the same sector in which they had registered (n=252, 89%), indicating substantial continuity of care, similar to findings reported by Dixit et al. [12]. Sectoral switching occurred in 31 (11%) of participants; however, the McNemar’s test did not show a significant net shift between sectors, suggesting bidirectional movement rather than systematic preference for one sector. Previous studies that have studied this switching, such as Khumukcham et al. [4] and Madhusoodanan et al. [2], have reported reasons similar to our study. Women registered initially in the public facilities due to accessibility and cost, but moved to private facilities for delivery due to perceived quality, provider availability, and trust. Women registering in private facilities reportedly returned to public facilities for cost containment and assured admission.

Consistent with existing literature, the cesarean section rate at the community level was high at 43% (121 deliveries), and significantly higher in private sector deliveries (n=56, 53% vs. n=65, 37%), despite similar indications across sectors. In Peru, Moquillaza-Alcantra et al. [24] reported higher cesarean section rates in private and insured facilities, often associated with higher socioeconomic status and institutional practices rather than medical necessity. In India, Khumukcham et al. [4] and Bhatia et al. [10] demonstrated a disproportionately rapid rise in cesarean section deliveries in the private sector over time, with minimal variation in indications between sectors, implicating non-clinical drivers such as provider preference, institutional incentives, and defensive medicine. The substantially higher OOPE associated with private and cesarean section deliveries highlights the risk of catastrophic health expenditure [4,9], emphasizing the need for future research on distress financing among urban poor households.

Regarding neonatal outcomes, birth weight, and early initiation of breastfeeding did not differ significantly by sector. However, public sector facilities demonstrated significantly higher coverage of birth vaccination and exclusive breastfeeding at discharge, reflecting stronger adherence to standardized newborn care protocols and breastfeeding promotion driven by program-based monitoring and accountability mechanisms. In contrast, in the private sector, birth-dose vaccination was often unavailable. It required referral to government health posts, leading to delays in timely Hepatitis-B administration, as also reported by Kumar et al. [25]. Additionally, higher cesarean section rates in private facilities may have adversely influenced breastfeeding practices. Previous studies have also reported better implementation of preventive newborn care in public facilities and lower rates of timely or exclusive breastfeeding in private settings, particularly in urban areas [4,12,26]. Although neonatal complications were more frequently reported following private sector deliveries, this difference was not statistically significant and likely reflects the higher burden of high-risk pregnancies and cesarean sections in private facilities rather than differences in neonatal care quality per se.

Taken together, these findings highlight the complementary roles of public and private sector maternal health services in urban slums. Public facilities continue to provide affordable, protocol-driven care with stronger postnatal practices and lower costs. In contrast, private facilities are more frequently used by women with higher socioeconomic status and clinical complexity, offering greater access to diagnostics and specialist care, albeit at higher costs and with higher intervention rates.

This study has certain limitations. As a cross-sectional study, causal relationships between sectoral choice, care practices, and delivery characteristics cannot be established. Information on ANC utilization, delivery practices, and OOPE was partly self-reported. It may be subject to recall bias, although verification against available medical records was undertaken to minimize this bias. Since sampling was undertaken rather than complete enumeration, selection bias cannot be entirely ruled out. A detailed evaluation of financial coping mechanisms such as borrowing, asset sale, taking loans, or long-term financial distress was not undertaken. Therefore, the extent of catastrophic health expenditure and sustained financial hardship could not be determined. Future studies should incorporate this to better understand the economic implications of sectoral choice.

Based on the study findings, several context-specific strategies are recommended. First-trimester registration in the public sector can be further strengthened through active facilitation by frontline health workers and by regularly updating the pregnancy registration targets based on the population size. Universal issuance and use of the MCP card across both public and private sectors must be ensured, given its central role in health education, counselling, monitoring pregnancy complications, and receiving monetary incentives under the Pradhan Mantri Matru Vandana Yojana [27]. A voucher-based system for first-trimester ultrasonography can be introduced to improve the ultrasonography coverage in the public sector, with which we can retain the woman’s registration in the public sector, while getting the ultrasonography done from accredited private centers at subsidized rates, or free of cost. This has already been successfully implemented for obtaining chest X-ray services under the National Tuberculosis Elimination Programme in Mumbai [28]. Currently, there are no private hospitals empaneled under the Pradhan Mantri Jan Arogya Yojana [29] or the Mahatma Jyotiba Phule Jan Arogya Yojana (State scheme) [30] within one hour of the field practice area. Empanelment of private maternity hospitals can help reduce OOPE for delivery care for eligible families. Birth immunization coverage in private facilities was relatively low due to fixed immunization days, unavailability of vaccines, and cost considerations. To improve this, a public-private partnership model can be tried. This was successfully implemented in Tripura [31], where a conveniently located Government hospital was selected as the hub providing vaccines and logistics daily to private nursing homes to ensure immunization of all newborns within 24 hours of delivery. Finally, the Public Health Department can institute a system of routine cesarean section audits across both sectors to promote rational obstetric practices.

## Conclusions

This community-based study in an urban slum of Mumbai suggests that the public sector was a more frequent entry point for maternal health care than the private sector. Private sector registration was associated with higher maternal education, prior cesarean section, and pre-existing medical disorders, suggesting that perceived clinical risk and awareness play a greater role than other sociodemographic and obstetric factors in determining the choice of sector for pregnancy registration. ANC coverage and process indicators were uniformly high across both sectors, challenging assumptions of inferior public-sector ANC. Public facilities showed stronger program linkage, and private facilities offered better access to early diagnostics. Sector switching was low and bidirectional, driven by factors such as perceived quality, waiting times, cleanliness of facilities, referral for complications, and cost considerations. However, deliveries in the private sector had significantly higher cesarean rates and OOPE, while public facilities performed better in newborn immunization and exclusive breastfeeding at discharge. Strengthening coordination, standardizing obstetric practices, and reducing unnecessary interventions are essential to improve continuity, equity, and financial protection in urban maternal health care.
